# Heparin-binding protein-enhanced quick SOFA score improves mortality prediction in sepsis patients

**DOI:** 10.3389/fmed.2022.926798

**Published:** 2022-08-11

**Authors:** Xiaotong Han, Qingli Dou, Yimin Zhu, Peng Ling, Yi-Hsuan Shen, Jiangping Liu, Zhongwei Zhang, Yucheng Zhou, Maiying Fan, Sih-Shiang Huang, Chien-Chang Lee

**Affiliations:** ^1^Clinical Research Center for Emergency and Critical Care in Hunan Province, Hunan Provincial People’s Hospital, The First Affiliated Hospital of Hunan Normal University, Changsha, China; ^2^The People’s Hospital of Baoan Shenzhen, Shenzhen, China; ^3^The Second Affiliated Hospital of Shenzhen University, Shenzhen, China; ^4^Institute of Emergency Medicine, Hunan Provincial Key Laboratory of Emergency and Critical Care Metabonomics, Hunan Provincial People’s Hospital (The First-Affiliated Hospital of Hunan Normal University), Changsha, China; ^5^Department of Critical Care Medicine, Shaoyang Central Hospital, Shaoyang, China; ^6^Department of Family Medicine, Taipei City Hospital, Taipei, Taiwan; ^7^Department of Emergency Medicine, University of Hong Kong-Shenzhen Hospital, Shenzhen, China; ^8^Department of Emergency Medicine, National Taiwan University Hospital, Taipei, Taiwan; ^9^Center of Intelligent Healthcare, National Taiwan University Hospital, Taipei, Taiwan

**Keywords:** sepsis, qSOFA score, heparin-binding protein, mortality, risk stratification, web calculator conceptualization, clinical data collection, data curation

## Abstract

**Purpose:**

The Quick Sequential Organ Failure Assessment (qSOFA) score proposed by Sepsis-3 as a sepsis screening tool has shown suboptimal accuracy. Heparin-binding protein (HBP) has been shown to identify early sepsis with high accuracy. Herein, we aim to investigate whether or not HBP improves the model performance of qSOFA.

**Methods:**

We conducted a multicenter prospective observational study of 794 adult patients who presented to the emergency department (ED) with presumed sepsis between 2018 and 2019. For each participant, serum HBP levels were measured and the hospital course was followed. The qSOFA score was used as the comparator. The data was split into a training dataset (*n* = 556) and a validation dataset (*n* = 238). The primary endpoint was 30-day all-cause mortality.

**Results:**

Compared with survivors, non-survivors had significantly higher serum HBP levels (median: 71.5 ng/mL vs 209.5 ng/mL, *p* < 0.001). Serum level of HBP weakly correlated with qSOFA class (*r*^2^ = 0.240, *p* < 0.001). Compared with the qSOFA model alone, the addition of admission HBP level to the qSOFA model significantly improved 30-day mortality discrimination (AUC, 0.70 vs. 0.80; *P* < 0.001), net reclassification improvement [26% (CI, 17–35%); *P* < 0.001], and integrated discrimination improvement [12% (CI, 9–14%); P < 0.001]. Addition of C-reactive protein (CRP) level or neutrophil-to-lymphocyte ratio (NLR) to qSOFA did not improve its performance. A web-based mortality risk prediction calculator was created to facilitate clinical implementation.

**Conclusion:**

This study confirms the value of combining qSOFA and HBP in predicting sepsis mortality. The web calculator provides a user-friendly tool for clinical implementation. Further validation in different patient populations is needed before widespread application of this prediction model.

## Introduction

Sepsis continues to be a major global health concern with the possibility of serious short and long-term complications (1). Despite increased clinical awareness, expedited administration of antibiotics and intravenous fluids, and advances in technology for organ function support, the mortality rate remains as high as 35% in severe sepsis. The Emergency Department (ED) plays an important role in sepsis care as the majority of sepsis patients are admitted to the hospital through the ED. Approximately 25% of ED sepsis patients’ progress to severe sepsis or septic shock within 72 h of presentation, highlighting the importance of early identification of high risk patients who would benefit from early intervention (2, 3). Early initiation of evidence-based sepsis bundle care has been associated with improved outcomes (4).

According to Sepsis-3 definition, sepsis is life-threatening organ dysfunction caused by a dysregulated host response to infection. Life-threatening organ dysfunctions are quantified by a change in the Sequential Organ Failure Assessment (SOFA) score by 2 or more points. Because SOFA score is not routinely calculated outside the ICU, a simplified version called the quick SOFA (qSOFA) score was developed for non-ICU settings, including the ED (5). qSOFA is used as a bedside assessment tool where patients with 2 or more should be further evaluated for sepsis. Since the introduction of qSOFA, more than 40 validation studies consisting of more than 400,000 patients have been conducted. A recent meta-analysis, however, showed that the accuracy of qSOFA was suboptimal with a pooled sensitivity of only 0.48 (95% CI: 0.41–0.55) (6–8).

Laboratory markers such as C-reactive protein (CRP), neutrophil to lymphocyte ratio (NLR), and lactate have been widely used to aid in the diagnosis of sepsis in clinical settings. However, none of them adequately predict the outcome (9). Recently, heparin-binding protein (HBP), a 37-kDa protein in the polymorphonuclear leukocyte, has been shown to outperform other infectious biomarkers in predicting the risk of progression to sepsis in a large meta-analysis (10). HBP is rapidly released upon adhesion of leukocytes to endothelial cells and induces capillary leakage with microcirculatory dysfunction (11). The unique feature that distinguishes HBP from other inflammatory biomarkers is its ability to predict shock as early as 72 h before its onset (12), and its high correlation with organ dysfunction (13). Neither CRP nor lactate have demonstrated these features.

Despite the suboptimal accuracy of qSOFA, its simplicity and clinical utility justifies its use in the management of sepsis (5). One plausible explanation for the suboptimal accuracy of qSOFA may be that it lacks variables that could detect early pathophysiological changes in sepsis before vital signs deteriorate (14). Therefore, the aim of this study was to evaluate whether or not adding HBP to the qSOFA score improves its ability to predict in-hospital mortality. We conducted a prospective multicenter cohort study and compared the relative performance of qSOFA modified by HBP to qSOFA modified by either CRP or NLR.

## Materials and methods

### Study design and population

We performed a multicenter prospective cohort study at three tertiary-care urban medical centers in China and Taiwan. Shenzhen PoAn Hunan People’s Hospital, Hunan Provincial People’s Hospital, and National Taiwan University Hospital. Patients were enrolled prospectively from June 1, 2018 to December 31, 2019. Adult patients (≥20 years old) who presented to the ED with suspected systemic infection were eligible for inclusion. Systemic infection was defined as the presence of at least two signs of systemic inflammation and laboratory or radiologic evidence of infection. In addition, included patients must have had at least one blood culture drawn. Signs of systemic inflammation include fever (>38.3°C) or hypothermia (<36°C), tachycardia (heart rate >90 beats/min), tachypnea (respiratory rate >20 breaths/min or PaCO_2_ <32 mmHg), and leukocytosis (WBC >12,000 cells/mm^3^) or leukopenia (WBC <4,000 cells/mm^3^). Laboratory evidence of infection included signs of inflammation [e.g., CRP levels lower than 10 mg/L are considered normal. CRP greater than 10 mg/L indicates clinically significant inflammatory processes (15)], the presence of pathogenic microorganisms cultured from bodily fluid (e.g., urine), or the presence of a local abscess. Radiologic evidence of infection includes infection-related findings on plain X ray, ultrasound, computed tomography, or magnetic resonance imaging. Those excluded were those with known pregnancy, do-not-resuscitate orders, immunocompromised patients, neutropenic patients (ANC count < 500/mm^3^), or those who received heparin treatment within 72 h (as this may affect serum levels of HBP). Hematological malignancies, terminal cancers, cancers under chemotherapy or radiation, HIV infections, patients taking steroids or immunosuppressants are considered immunocompromised. Patients transferred from an outside hospital were also excluded. They were excluded from the study since they had already been treated and stabilized, thus not comparable to individuals who presented to the emergency department for the first time. This study was approved by the Institutional Review Boards of all institutions.

### Measurement of heparin-binding protein

Blood samples of eligible participants were collected in the ED and centrifuged at 2,200 g for 10 min. Serum levels of HBP were measured in a blinded manner with regard to the clinical condition and qSOFA of the patient at the time of blood draw. The concentrations of HBP were assayed in a single batch at three major sites using an enzyme immunoassay from JoinStar (Hangzhou, China), according to manufacturer’s instructions. Upon collection, samples were centrifuged and stored at −20°C refrigerator until measurement. Limit of detection is reported to be 5.9 ng/mL. Inter-assay coefficients of variation were measured in 11 replicates and were 11% at 21 ng/mL and 7% at 81 ng/mL. Serum levels of CRP at ED admission were determined by Aeroset 2.0 analyzer (Abbott Diagnostics, Santa Clara, CA, United States). The neutrophil to lymphocyte ratio (NLR) was calculated by dividing the neutrophil count by the lymphocyte count.

### Data collection

Physicians involved in the study collected patient data using a standardized instrument. Physiological and laboratory variables at time of ED admission were recorded. SIRS criteria variables included abnormal body temperature, tachycardia, tachypnea, and abnormal white blood cell count. Criteria for qSOFA included altered mental status (Glasgow coma scale ≤14), hypotension (systolic blood pressure ≤100 mmHg), and tachypnea (respiratory rate >22/min). For organ dysfunction, we adopted the CDC adult sepsis event criteria definition (16). In brief, we defined septic shock as Initiation of a new vasopressor infusion (norepinephrine, dopamine, epinephrine, phenylephrine, or vasopressin), respiratory failure as the need for invasive mechanical ventilation, acute kidney injury as doubling of serum creatinine or decrease by ≥ 50% of estimated glomerular filtration rate (eGFR) relative to baseline levels in 7 days or ≥ 0.3 mg/dL within 48 h, excluding patients with ICD-10 code for end-stage renal disease, acute hepatic dysfunction as total bilirubin ≥ 2.0 mg/dL and increase by 100% from baseline, acute hematological function as platelet count < 100 cells/μL and ≥ 50% decline from baseline (baseline must be ≥ 100 cells/μL), acute mental status change as Glasgow Coma Scale score of < 15 or a decrease in the score by at least 1 in those with pre-existing central nervous system disease. The source of infection was classified by the final discharge diagnosis of pneumonia, urinary tract infection, biliary tract infection, intra-abdominal infection, skin and soft tissue infection, bloodstream infection, and miscellaneous source of infection.

### Statistical analysis

Categorical variables were expressed as frequencies and compared using Fisher’s exact test or a Chi-squared test, as appropriate. Continuous variables were reported as median with interquartile range (IQR) and compared using Mann–Whitney *U*-test. We calculated the Spearman’s rank correlation coefficient and drew qSOFA score-stratified box plots to assess the correlations between three laboratory markers and clinical severity. We randomly split the data into a derivation cohort (70%) and a validation cohort (30%). We created three laboratory marker-modified models (qSOFA_NLR score, qSOFA_CRP score and qSOFA_HBP score) in the derivation cohort and validated the accuracy in the validation cohort. The serum levels of HBP were classified into tertile ordinal classes (0 for HBP under 41 ng/mL, 1 for HBP between 41 and 151 ng/mL, and 2 for HBP above 151 ng/mL), bringing qSOFA to a five-point scale. The new model still considers 2 qSOFA points positive. The cutoff level was determined empirically based on previous literature review and a restricted cubic spline analysis (10, 17). Following the best practice of presenting a clinical prediction model (18), we reported the discrimination and calibration of the three models. Discrimination was calculated by area under the receiver operating characteristic curve (AUC) and compared with a de Long test. Calibration was evaluated by a calibration plot and Brier score. Brier score checks the goodness of a predicted probability score.

Next, we conducted reclassification analysis to assess whether or not the biomarker-modified qSOFA models significantly reclassified patients into more appropriate risk categories. We divided all patients into three predicted mortality risk groups empirically: low risk (0% to less than 15%), moderate risk (15% to less than 35%), and high risk (35% or greater) and calculated the net reclassification improvement (NRI) and the integrated discrimination improvement (IDI). The NRI was calculated by summing the proportion of participants across risk categories whose estimated risk shifts in the correct direction minus the proportion of participants whose risk shifts in the incorrect direction. The IDI calculates the difference in discrimination slopes between the two models, thereby demonstrating the improvement in both discrimination and reclassification. Continuous NRI is a non-parametric analog of the IDI and equals twice the difference in probabilities of upward reclassification for events minus for non-events. The NRI estimated overall improvement in reclassification with the new model. The IDI estimated improvement in both discrimination and reclassification. We performed two sensitivity analyses to verify the robustness of the analysis. We calculated the categorical NRI using different risk categories (20%, 40%) and continuous NRI. The detailed methods for calculating NRI and IDI are presented in the Supplementary Material. Lastly, we developed a risk calculator using the Shiny package of R (Foundation for Statistical Computing, Vienna, Austria). We adhered to the transparent reporting of a multivariable prediction model for individual prognosis or diagnosis (TRIPOD) statement for reporting (18). All analyses were performed with SAS Version 9.4 (Cary, NC, United States) except for the NRI and IDI statistics which were performed with R. A 2-sided *p*-value < 0.05 was considered significant.

## Results

### Study design and patient characteristics

**FIGURE 1:**
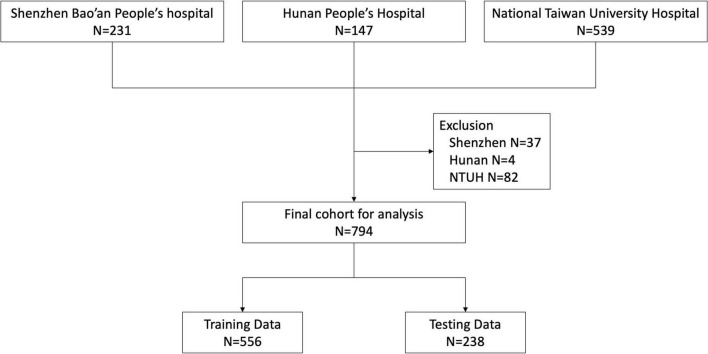
Flowchart of patient inclusion and exclusion.

During the study period, 231, 147, and 539 patients were enrolled from Shenzhen Bao’an People’s Hospital, Hunan People’s Hospital and National Taiwan University Hospital, respectively. We excluded 37 patients from Shenzhen, 4 from Hunan and 82 from NTUH according to our exclusion criteria. Ultimately, 794 patients were eligible for analysis (Figure 1). We classified the patient cohort into three severity groups: survivors (patients hospitalized without events), critically-ill (patients admitted to the ICU but survived for more than 30 days), and non-survivors (patients who died within 30 days of hospital admission). Compared to survivors, critically-ill and non-survivors were older and more likely to have developed acute organ dysfunction or shock. In addition, critically-ill or non-surviving patients more frequently had infections of the lower respiratory tract, abdomen, and bloodstream. In contrast, surviving patients more frequently had infections of the urinary tract, biliary tract, and skin and soft tissue. Vital signs and laboratory data were also correlated with the severity groups. Comparison of patient characteristics across three groups is summarized in Table 1.

**TABLE 1:** Characteristics of the study patients, stratified by three different severity groups.

	Surviving patients (*N* = 350)	Critically-ill patients (*N* = 265)	Non-surviving patients (*N* = 179)	*P*-value
Age (years)	62 (52, 77)	67 (52, 77)	67 (53, 80)	0.4206
Male gender	214 (61.1%)	171 (64.5%)	128 (71.5%)	0.0618
Severe sepsis	226 (66.7%)	239 (91.2%)	153 (91.6%)	< 0.0001***
Septic shock	31 (8.9%)	57 (21.7%)	83 (46.9%)	< 0.0001***
**Source of infection**				
Pneumonia	114 (32.6%)	176 (66.4%)	107 (59.8%)	< 0.0001***
Urinary tract infection	64 (18.3%)	28 (10.6%)	13 (7.3%)	0.0006***
Biliary tract infection	51 (14.6%)	9 (3.4%)	11 (6.2%)	< 0.0001***
Intra-abdominal infection	89 (25.4%)	42 (15.1%)	27 (15.9%)	0.0024**
Skin and soft tissue infection	34 (9.7%)	5 (1.9%)	8 (4.5%)	0.0002**
Bloodstream infection	7 (2.0%)	26 (9.8%)	22 (12.3%)	< 0.0001***
Miscellaneous	12 (3.4%)	33 (12.5%)	21 (11.7%)	< 0.0001***
**Organ dysfunction**				
Acute respiratory failure	95 (27.1%)	196 (74.0%)	130 (72.6%)	< 0.0001***
Cardiovascular dysfunction	82 (23.4%)	123 (46.4%)	119 (66.5%)	< 0.0001***
Acute renal dysfunction	61 (17.4%)	111 (41.9%)	91 (50.8%)	< 0.0001***
Acute hepatic dysfunction	52 (14.9%)	78 (29.4%)	53 (29.6%)	< 0.0001***
Acute hematologic dysfunction	37 (10.6%)	62 (23.4%)	47 (26.3%)	< 0.0001***
Altered mental status	62 (17.7%)	120 (45.3%)	127 (71.0%)	< 0.0001***
**qSOFA variables**				
GCS	15 (15, 15)	15 (11, 15)	11 (5, 15)	< 0.0001***
SBP (mmHg)	130 (110, 148)	123 (105,145)	114 (94,135)	< 0.0001***
Respiratory rate (min^–1^)	20 (18, 20)	22 (20, 27)	22 (20, 26)	< 0.0001***
**Laboratory markers**				
WBC count (10^3^/mm^3^)	10.20 (6.90, 14.06)	10.95 (7.11, 14.73)	12.36 (8.75, 17.31)	0.0003***
Platelet count (10^3^/mm^3^)	217 (147, 282.)	174 (114, 261)	194 (110, 267)	0.0008*
HBP (ng/mL)	71.5 (28.7, 156.6)	73.8 (36.2, 139.5)	209.5 (116.0, 286.2)	< 0.0001***
CRP (mg/L)	10.3 (6.6, 16.4)	48.4 (17.8, 108.4)	22.0 (11.3, 101.2)	< 0.0001***
NLR ratio	8.0 (4.5, 13.6)	11.2 (5.8, 21.2)	7.8 (4.5, 15.2)	0.0002***

***Means *p*-value < 0.001, ** means *p*-value < 0.01, * means *p*-value < 0.05.

### Association between laboratory markers and organ dysfunction or qSOFA

**FIGURE 2:**
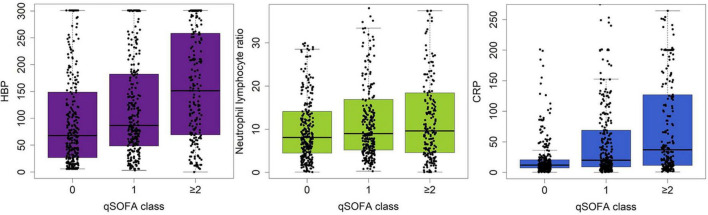
Boxplot showing the correlation between serum levels of HBP, CRP and NLR and the qSOFA class (0, 1, ≥ 2). HBP has the highest correlation with qSOFA class, followed by CRP or NLR.

Table 2 details the discrimination of three different markers on six sepsis-associated six acute organ dysfunctions. HBP had high discrimination for all six organ dysfunctions. CRP had moderate discrimination and NLR had the least discrimination for acute organ dysfunctions. Figure 2 shows the boxplots of three laboratory markers stratified by qSOFA score class (0, 1, ≥ 2). CRP has the highest correlation with qSOFA class (*r*^2^ = 0.30, *p* < 0.001), followed by HBP (*r*^2^ = 0.240, *p* < 0.001). NLR did not significantly correlate with the qSOFA class (*r*^2^ = 0.063, *p* = 0.076). Both CRP and HBP were weakly correlated with qSOFA.

**TABLE 2:** Discrimination of HBP, CRP, and NLR on acute organ dysfunction.

AUC with 95% Confidence Intervals	HBP	CRP	NLR
Acute respiratory failure	0.79 (0.76–0.83)	0.65 (0.62–0.70)	0.65 (0.60–0.69)
Cardiovascular dysfunction	0.80 (0.77–0.84)	0.70 (0.66–0.74)	0.68 (0.63–0.73)
Acute renal dysfunction	0.79 (0.76–0.83)	0.65 (0.61–0.70)	0.63 (0.58–0.68)
Acute hepatic dysfunction	0.78 (0.74–0.81)	0.61 (0.56–0.66)	0.55 (0.50–0.60)
Acute hematologic dysfunction	0.78 (0.74–0.81)	0.60 (0.55–0.65)	0.57 (0.52–0.62)
Altered mental status	0.82 (0.78–0.85)	0.73 (0.69–0.77)	0.73 (0.69–0.77)

### Biomarker-enhanced qSOFA models: Discrimination and calibration

**FIGURE 3:**
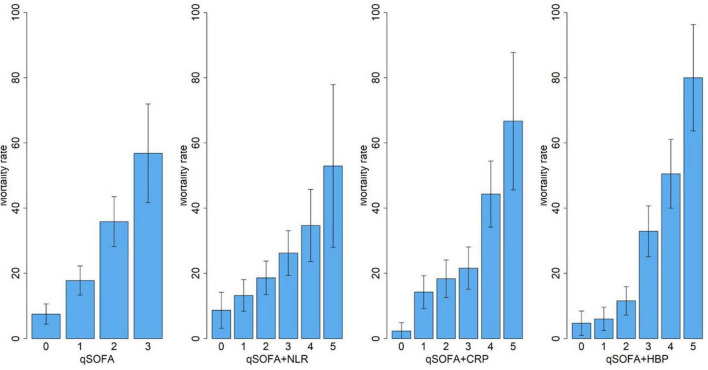
Calibration of qSOFA, qSOFA-NLR, qSOFA-CRP and qSOFA-HBP. Hosmer-Lemeshow Chi-square is 0.90 for qSOFA score only (*P* = 0.34),3.64 for qSOFA_HBP (*P* = 0.30), 2.52 for qSOFA_NLR score (*P* = 0.47), and 7.70 for qSOFA_CRP score (*P* = 0.05).

We built three biomarker-enhanced qSOFA models: qSOFA_NLR, qSOFA_CRP and qSOFA_HBP. In the validation cohort, the qSOFA_HBP score had the highest AUC (0.80, 95% CI, 0.73–0.87), followed by qSOFA (0.70, 95% CI, 0.62–0.77), qSOFA_CRP (0.66, 95% CI, 0.58–0.74) and qSOFA_NLR (0.61, 95%CI: 0.53–0.69) (Table 3 and Supplementary Figure 1). Compared to qSOFA alone, the addition of HBP to qSOFA significantly improved sepsis mortality discrimination (de Long test *P* < 0.001). Visual examination of observed versus model-predicted 30-day mortality suggested improved agreement with the qSOFA_HBP model (Brier score: 0.134), followed by qSOFA (Brier score: 0.155), qSOFA_CRP: (Brier score: 0.160), and qSOFA-NLR: (Brier score: 0.169) (Supplementary Figure 2). Figure 3 demonstrates the calibration plot of qSOFA alone, qSOFA_NLR, qSOFA_CRP and qSOFA_HBP. Hosmer–Lemeshow Chi−square for qSOFA score only, qSOFA_HBP, qSOFA_NLR and qSOFA_CRP is 0.90 (*P* = 0.34), 3.64 (*P* = 0.30), 2.52 (*P* = 0.47), and 7.70 (*P* = 0.05) respectively.

**TABLE 3:** Discrimination of qSOFA and modified qSOFA prediction models in derivation and validation datasets.

AUC	Derivation dataset (*n* = 556)	Validation (*n* = 238)
qSOFA only	0.71 (0.67–0.76)	0.70 (0.62–0.77)
qSOFA + NLR	0.63 (0.58–0.68)	0.61 (0.53–0.69)
qSOFA + CRP	0.70 (0.65–0.75)	0.66 (0.58–0.74)
qSOFA + HBP	0.80 (0.75–0.84)	0.80 (0.73–0.87)

### Mortality risk reclassification

With the addition of HBP to the qSOFA model, the difference between the proportion of non-survivors who moved up a risk category and the proportion who moved down, plus the difference between the proportion of survivors who moved down a risk category and the proportion who moved up (net reclassification improvement), was 26% (CI, 17–35%; *P* < 0.0001). This improvement in risk reclassification was largely driven by enhanced prediction among surviving patients (24%) and to a lesser extent by reclassification of non-surviving patients (2%) (Supplementary Table 1). The difference in average predicted probability of mortality between surviving and non-surviving patients (integrated discrimination improvement) significantly increased after adding HBP to the qSOFA model (12%, 95%CI, 9–14%, *P* < 0.0001). Sensitivity analysis using a different cutoff (20%/40%) to define the risk category or using a category-free continuous NRI also showed a significant improvement in mortality risk reclassification (Table 4).

**TABLE 4:** Net reclassification improvement (NRI) with HBP-modified qSOFA score using 15 and 35% or 20% and 40% as cutoffs to define patient subgroups at low, intermediate, or high risk.

	NRI (95% CI)	*P*-value	IDI (95% CI)	*P*-value
**qSOFA_HBP vs. qSOFA (endpoint: 30-day mortality)**				
NRI (15%/35%)	25.7% (16.7%–34.6%)	< 0.0001***	0.12 (0.09–0.14)	< 0.0001***
NRI (20%/40%)	15.7% (8.1%–23.3%)	< 0.0001***	0.12 (0.09–0.14)	< 0.0001***
NRI (continuous)	82.4% (66.9%–97.9%)	< 0.0001***	0.12 (0.09–0.14)	< 0.0001***

Category-free NRI was also calculated.

The analysis is based on all patients.

CI, confidence interval; NRI, net reclassification index, IDI, integrated discrimination improvement.

### Web-based calculator

The online mortality risk calculator developed based on our study is available at: (https://stacysu.shinyapps.io/Mortality_Prediction_Probability/) (Supplementary Figure 3). It illustrates how qSOFA and HBP affect mortality estimates. Clinical users can input data for Glasgow coma scale, systolic blood pressure, respiratory rate, and serum HBP level to calculate predicted 30-day mortality.

## Discussion

As the list of biomarkers and validated clinical scores for sepsis continues to grow the potential value of combining these diagnostic tools is of particular interest. In this prospective multicenter study of sepsis patients, we demonstrate that incorporating serum HBP levels with qSOFA score at time of ED admission significantly improves classification. We found that HBP predicts sepsis-related acute organ dysfunction and may improve the accuracy of qSOFA scores. In our study, both CRP and NLR failed to improve predictive accuracy of qSOFA.

Few studies have investigated the value of combining clinical scoring systems and infection biomarkers in predicting sepsis mortality. Yu et al. showed that combining qSOFA and procalcitonin may significantly improve the performance of qSOFA score (19). Viallon et al. showed that combining SAPS-2, procalcitonin, lactate, and IL-6, could predict sepsis mortality with high accuracy (AUC 0.94) (20). However, this was a single center study without an independent sample validation. Furthermore, the SAPS-2 and SAPS-3 scores, which include 17 and 20 variables respectively, are complicated and not routinely available outside of the ICU (21). Mellhammar J et al. found that including HBP into qSOFA (additional 1 point for HBP > 30 ng/mL) significantly improved prediction of mortality in patients with suspected infection (20); the AUC improved from 0.70 (95%CI: 0.66–0.75) for qSOFA alone to 0.78 (95%CI: 0.74–0.82) for HBP modified qSOFA. Nevertheless, independent sample validation was not performed in their study, and prognostic information was lost because HBP was dichotomized. Per Sepsis-3, qSOFA is a standard tool for sepsis diagnosis and prognosis in settings outside the ICU. Although calculating qSOFA is simple and straightforward, its suboptimal predictive accuracy limits its use. Our work demonstrates how to quantitatively combine a clinically useful biomarker with a widely validated prediction rule. Our approach has the advantage of easy clinical implementation without needing to develop a more complicated new scoring system.

The physiological mechanism of HBP release in sepsis may offer insight into why HBP can provide incremental prognostic value to the qSOFA score. Heparin-binding protein (HBP), also known as azurocidin or CAP37, is a chemoattractant that activates neutrophils, T lymphocytes and monocytes, enhances cytokine release and phagocytosis, and induces vascular leakage (22). HBP is an inflammatory mediator released immediately upon neutrophil stimulation (23). Although most inflammatory processes involving neutrophil activation can induce HBP release, several bacteria, including *Streptococcus pyogenes, Staphylococcus aureus, and E. coli* were found to be potent inducers (24–26). Therefore, serum level of HBP is particularly elevated in bacterial infections that result in sepsis (27). It has been shown that HBP levels are significantly elevated in sepsis, urinary tract infections, bacterial skin and soft tissue infections, and bacterial meningitis (28, 29). Compared to common inflammatory biomarkers such as CRP, procalcitonin or IL-6, HBP is unique in that it induces vascular leakage (22), and therefore microcirculatory dysfunction, the hallmark of sepsis-induced organ dysfunction (30). Accordingly, our study, as well as previous ones, observed a correlation between serum level of HBP and acute kidney injury, respiratory failure, and circulatory failure (31, 32). In a recent systematic review and meta-analysis consisting of 3,868 patients, HBP demonstrated high specificity and sensitivity in predicting progression to sepsis in critically ill patients with a pooled sensitivity of 0.85 (95% CI, 0.79–0.90) and a pooled specificity of 0.91 (95% CI 0.82–0.96). In addition, HBP has been shown to be an important predictor of sepsis mortality with a sensitivity of 0.87 and specificity of 0.71 (33). Moreover, it has been shown that patients have elevated serum HBP levels up to 72 h before sepsis shock or organ dysfunction develop (34), which makes HBP a promising tool for the early detection of patients at risk of developing severe sepsis in the ED.

Results of this study should be interpreted in light of its strengths and limitations. To begin, strengths include the prospective multicenter cohort design and independent sample validation, as this may minimize risk of selection bias while maximizing generalizability. The rigorous statistical analysis ensures the robustness of the model. The web calculator increases the feasibility of clinical implementation. This study also has limitations. First, due to the observational nature, this study does not address whether or not the use of qSOFA_HBP as a risk prediction tool improves patient outcome in clinical practice. Second, due to the high cost for the central laboratory ELISA-based HBP measurement, HBP may be difficult to apply in rural areas. The recent development of point-of-care (POC) tests for HBP makes the wide application of HBP-modified qSOFA possible. Third, we used SIRS as the inclusion criteria to enroll study patients. Studies have shown SIRS has suboptimal sensitivity in identifying critical sepsis patients, especially elderly or immunosuppressed patients with fewer signs of inflammation. The benefit of combining HBP and qSOFA in this underrepresented patient population may need to be verified in future studies. Fourth, we did not exclude patients with renal function impairment, and it is plausible that sepsis-related acute kidney injury and subsequent continuous renal replacement therapy (CRRT) may confound HBP measurements and mortality outcome. However, it is notable that a recent study by Samuelsson showed that CRRT does not influence serum levels of HBP (35). Finally, evaluation of the relationship between the level of heparin binding protein and specific pathogens, was beyond the scope of the study. Further studies are required to determine the reliability of HBP as a marker for all sepsis-inducing microbes.

In conclusion, this study confirms the value of combining qSOFA and HBP in sepsis mortality prediction. The web calculator provides a user-friendly tool for convenient and accessible clinical implementation. Further validation in different patient populations is needed before widespread application of this tool.

## Data availability statement

The raw data supporting the conclusions of this article will be made available by the authors, without undue reservation.

## Ethics statement

The studies involving human participants were reviewed and approved by Shenzhen Baoan Hunan People’s Hospital (BYL 20190801), Hunan Provincial People’s Hospital (HNSRMYY REC2018-40), National Taiwan University Hospital (NTUH 201712161RINA). The patients/participants provided their written informed consent to participate in this study.

## Author contributions

XH and QD: clinical data collection, methodology, data curation, and writing—reviewing and editing. YiZ and PL: clinical data collection and writing—reviewing and editing. Y-HS: writing—original draft, project administration, and writing—reviewing and editing. JL and ZZ: clinical data collection, data curation, project administration, and writing—reviewing and editing. YuZ, MF, and S-SH: clinical data collection and writing—reviewing and editing. C-CL: conceptualization, methodology, clinical data collection, data curation, statistical analysis, writing—original draft, reviewing and editing, supervision, full access to all of the data in the study, responsibility for the integrity of the data, and the accuracy of the data analysis. All authors contributed to the article and approved the submitted version.

## Abbreviations

SOFA: Sequential Organ Failure Assessment
qSOFA: Quick Sequential Organ Failure Assessment
ED: Emergency department
HBP: Heparin-binding protein
CRP: C-reactive protein
NLR: Neutrophilto-lymphocyte ratio
ICU: Intensive care unit
WBC: White blood cell
ANC: Absolute neutrophil count
IQR: Interquartile range
AUC: Areaunder curve
NRI: Net reclassification improvement
IDI: Integrated discrimination improvemen
CRRT: Continuous renal replacement therapy
