# Supplementary material for: Heparin-binding protein-enhanced quick SOFA score improves mortality prediction in sepsis patients
Source: Front Med (Lausanne). 2022 Aug 11;9:926798. doi: 10.3389/fmed.2022.926798 (PMC9402998; doi:10.3389/fmed.2022.926798)
Supplement: Supplementary file 1 [file Data_Sheet_1.PDF]

Supplementary figure 1: ROC curves and corresponding areas under the curves of qSOFA models and modified qSOFA models in derivation and validation data sets

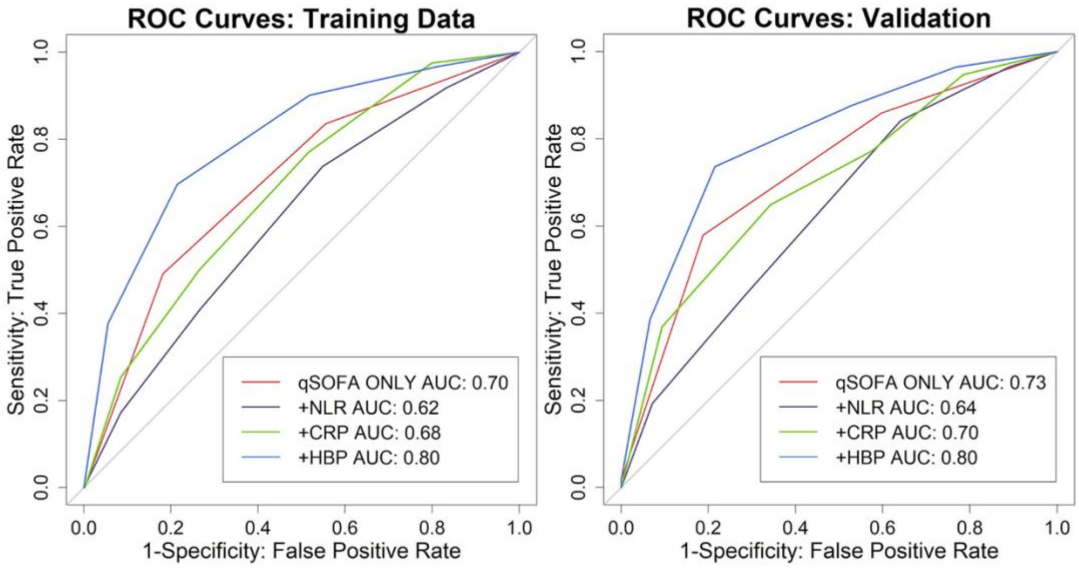

Supplementary figure 2: Predicted risk vs. observed risk of death. Risk predicted by the qSOFA score only (A), qSOFA score adjusted by HBP (B), qSOFA score adjusted by CRP (C), qSOFA score adjusted by NLR (D).

(A)

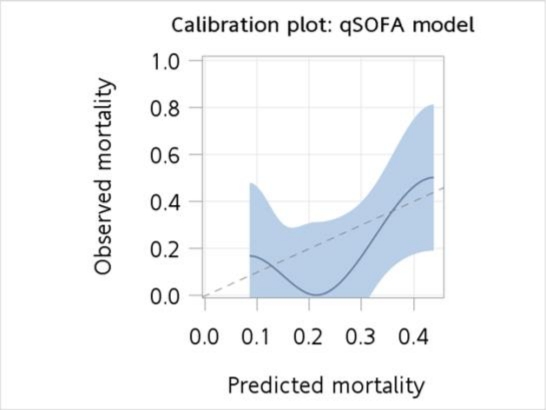

(B)

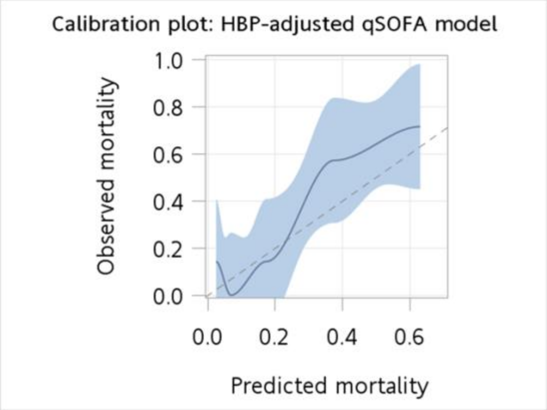

(B)

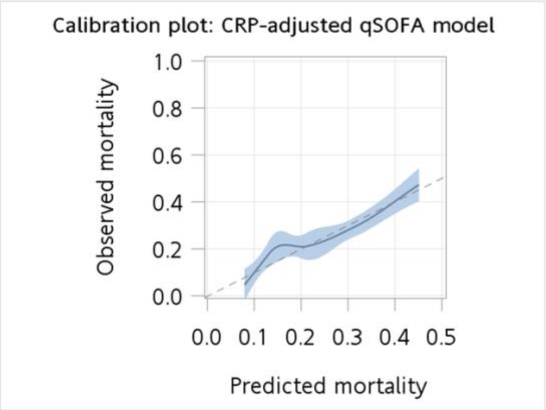

(D)

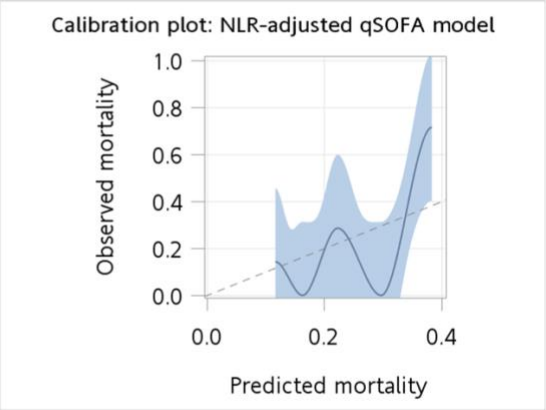

Supplementary figure 3. The qSOFA_HBP web calculator predicts 30-day mortality risk for ED patients with presumed systemic infection

(https://stacysu.shinyapps.io/Mortality_Prediction_Probability/)

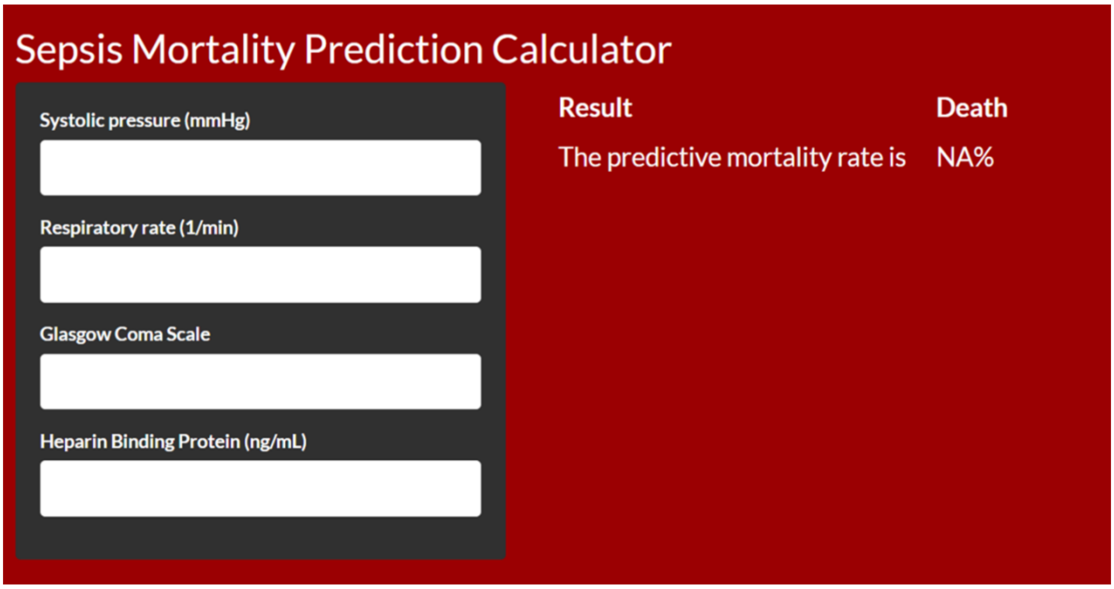

Supplemental table 1: Reclassification across pre-defined risk thresholds in the validation cohort using the algorithm for qSOFA score adjusted by heparin binding protein (NRI)

	qSOFA_HBP model			
qSOFA model	<15%	15–35%	>35%	All
Surviving patients, NRI = 0.24				
<15%	205	61	0	266
15–35%	89	92	55	236
>35%	0	37	76	113
All	294	190	131	615
Nonsurviving patients, NRI = 0.02				
<15%	14	14	0	28
15–35%	5	17	36	58
>35%	0	2	91	93
All	52	59	68	179

Supplementary description of calculating net reclassification improvement (NRI) and integrative discriminative improvement (IDI)

The NRI was calculated by summing the proportion of participants across risk categories whose estimated risk shifts in the correct direction minus the proportion of participants whose risk shifts in the incorrect direction. The IDI calculates the difference in discrimination slopes between the two models, thereby demonstrating the improvement in both discrimination and reclassification. Continuous NRI is a non-parametric analogue of the IDI and equals twice the difference in probabilities of upward reclassification for events minus for non-events.
